# The Feasibility and Functional Performance of Ternary Borate-Filled Hydrophilic Bone Cements: Targeting Therapeutic Release Thresholds for Strontium

**DOI:** 10.3390/jfb8030028

**Published:** 2017-07-14

**Authors:** Kathleen MacDonald, Richard B. Price, Daniel Boyd

**Affiliations:** 1 School of Biomedical Engineering, Dalhousie University, Halifax, NS B3H 1X7, Canada; kathleen.n.macdonald@dal.ca; 2 Department of Dental Clinical Sciences, Dalhousie University, Halifax, NS B3H 1X7, Canada; richard.price@dal.ca; 3 Department Applied Oral Sciences, Dalhousie University, Halifax, NS B3H 1X7, Canada

**Keywords:** composite resin cement, bone cement, borate glass, strontium, therapeutic inorganic ions, hydroxyethyl methacrylate

## Abstract

We examine the feasibility and functionality of hydrophilic modifications to a borate glass reinforced resin composite; with the objective of meeting and maintaining therapeutic thresholds for Sr release over time, as a potential method of incorporating antiosteoporotic therapy into a vertebroplasty material. Fifteen composites were formulated with the hydrophilic agent hydroxyl ethyl methacrylate (HEMA, 15, 22.5, 30, 37.5 or 45 wt% of resin phase) and filled with a borate glass (55, 60 or 65 wt% of total cement) with known Sr release characteristics. Cements were examined with respect to degree of cure, water sorption, Sr release, and biaxial flexural strength over 60 days of incubation in phosphate buffered saline. While water sorption and glass degradation increased with increasing HEMA content, Sr release peaked with the 30% HEMA compositions, scanning electron microscope (SEM) imaging confirmed the surface precipitation of a Sr phosphate compound. Biaxial flexural strengths ranged between 16 and 44 MPa, decreasing with increased HEMA content. Degree of cure increased with HEMA content (42 to 81%), while no significant effect was seen on setting times (209 to 263 s). High HEMA content may provide a method of increasing monomer conversion without effect on setting reaction, providing sustained mechanical strength over 60 days.

## 1. Introduction

Strontium (Sr) has been demonstrated in various in vivo models to improve bone mass [1] and strength [2], leading to its subsequent use as a treatment for osteoporosis [3]. While oral Sr therapy (i.e., Servier Laboratories’ strontium ranelate), has demonstrated up at a 49% reduction in vertebral fracture rate [4], early clinical trials predated a full understanding of the mechanism-of-action of strontium on bone [5]. Further research has since identified both localized and systemic effects associated with Sr [6,7]. Beyond local efficacy, strontium also demonstrates accumulation in bone tissue, potentially providing sustained therapeutic effect following dosing [8,9]. The discovery of its localized efficacy (and underlying mechanisms) has consequently lead to the investigation and development of therapeutic biomaterials for targeted local release of Sr ions [10,11,12].

From a design input standpoint, it is now recognized that the mechanism-of-action of Sr ions on bone metabolism relates to an uncoupling effect on osteoblast and osteoclast metabolism, which acts through the calcium sensing receptor in both cell lines. Specifically, increases in osteoblast proliferation and activity have been reported to occur at minimum Sr levels ranging from 8.7 mg/L [6] to 35 mg/L [13] while osteoclast inhibition occurs at higher Sr concentrations of 88 mg/L under in vitro conditions [6,14]. Strontium also requires a sufficient duration of exposure, of over 20 days before the full benefit is achieved [6,15]. While a minimum effective Sr dose is difficult to establish for localized release, it is agreed that the effective dosage window is large due to its relatively low toxicity [16,17]. Accordingly, localized release systems may target between 8.7 mg/L and 88 mg/L of Sr, the therapeutic threshold for osteoblasts and osteoclasts respectively.

The development of Sr-releasing injectable bone cements for the augmentation of osteoporotic vertebral compression fractures, is an area of particular interest in the literature [18,19,20,21,22,23,24]. Despite predating much of the clinical evidence, and the detailed mechanistic understanding of Sr as an antiosteoporotic agent, such materials have provided enhanced bone integration relative to controls [24,25,26]. Nonetheless, these studies lacked the guidance of accepted therapeutic threshold limits required for altered bone metabolism (including temporal dependencies), and, as such, the assessment of strontium release relative to these levels has not been widely studied. To develop an effective Sr releasing biomaterial capable of exerting an anti-osteoporotic effect, concept materials must be approached from a drug-device combination development standpoint. It must be engineered to provide both: (i) the necessary Sr release levels and (ii) maintain this therapeutic level over the duration required to achieve optimized therapeutic efficacy [6,15]. 

A Sr releasing anti-osteoporotic cement must also maintain the characteristics necessary of a vertebroplasty material, including sufficient sustained mechanical strength, injectability, and biocompatibility [27]. Bisphenol glycidyl methacrylate (Bis-GMA) based composite resin cements (e.g., Cortoss^®^, Orthovita, Malvern, PA, USA) have demonstrated improved biocompatibility relative to conventional poly(methyl methacrylate) (PMMA)cements for vertebral body augmentation, while simultaneously proving a method of therapeutic ion release from the inorganic filler phase [28]. As such Bis-GMA based composite resins present an ideal base for the development of a vertebroplasty material capable of releasing Sr at a therapeutic level. Previous studies aiming to release therapeutic agents from such dimethacrylate cements (both in dental and orthopedic applications) have utilized hydrophilic monomer additions to increase water sorption, and therefore mediate subsequent ion (or drug) release in a controlled fashion [29,30,31]. For example the incorporation of hydroxyl ethyl methacrylate (HEMA) into a bisphenol glycidyl methacrylate (Bis-GMA), triethylene glycol dimethacrylate (TEG DMA) system has been shown to double the total ion release from composite dental cements [32]. 

While this approach has proven to be effective in increasing water sorption and release, the filler phase of composite cements is frequently based on phosphosilicate glasses, which show limited degradation kinetics (that decay rapidly over time) [33], and are thus unlikely to provide sufficient sustained release. While phosphosilicate bioactive glasses show a rapid decay in ion release, borate based glasses can be designed to release ions both through leaching as well as through the direct hydrolysis of the glass network, allowing for sustained ion release kinetics [34]. A previously investigated borate glass showed preferential, diffusion controlled release of Sr with 38% percent of initial Sr content released over 60 days [35]. While they present many advantages for use in therapeutic ion delivery, fully borate based bioactive glasses cannot be silanized, potentially weakening the filler matrix interface [36]. This effect may be magnified by the effect of adding HEMA, which has previously been reported to decrease mechanical strength by as much as 40% [37].

Arising from these questions, and limitations, this study has been designed to investigate the degradation kinetics of a series of borate-glass composite resin cements to assess the feasibility and functionality of such a system as a therapeutic Sr releasing biomaterial for use as a vertebroplasty material. To achieve this goal, a well-characterized borate glass with Fickian diffusion-controlled Sr release kinetics [35] has been selected for use as a reinforcing agent, in a Bis-GMA, TEG DMA, HEMA blend resin phase. This study is designed to assess the effect of both resin hydrophilicity as well as glass filler content (55–65% of total cement weight) on cement handling characteristics, strength, water sorption, and Sr release over a 60 day time frame with a view to establishing Sr release levels capable of achieving the 88 mg/L required for uncoupling, for over 20 days to achieve maximum therapeutic efficacy.

## 2. Results

### 2.1. Cement Handling Properties

**Figure 1:**
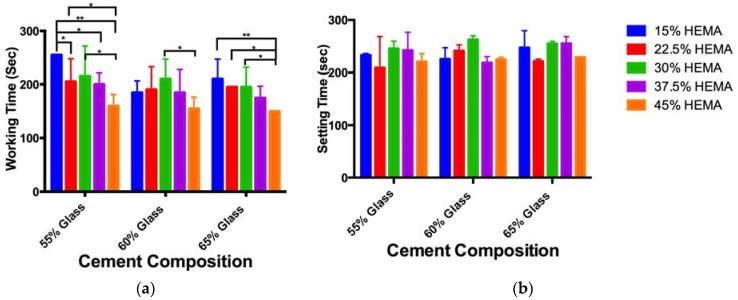
(**a**)Cement working time, and (**b**) cement setting time, significant differences are marked with “*” (*p* < 0.05) or “**” (*p* < 0.01).

The working times for the cements examined (Table 1) ranged from 150 to 255 s (Figure 1a), cement setting time ranged from 209 to 263 s (Figure 1b). While decreases in working time with increasing HEMA content were seen for some cement compositions (significant difference between composition A1 and B1, D1, E1; composition B1 and E1; composition C1 and E1; composition C2 and E2; composition E3 and A3, B3, C3), no overall trend was noted. No significant differences were seen between cement setting times.

### 2.2. ATR-FTIR Degree of Conversion

**Figure 2:**
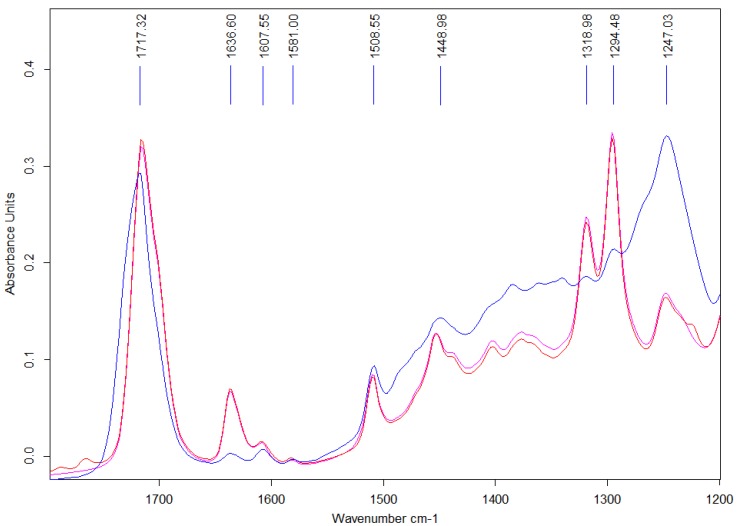
Representative Attenuated total reflectance Fourier transform infrared (ATR-FTIR) spectra collected on unset cement pastes (red and pink traces for paste one and two respectively), and set cement samples (blue trace) of cement composition A1 (15% hydroxyl ethyl methacrylate (HEMA), 55% glass).

Attenuated total reflectance Fourier transform infrared (ATR-FTIR) spectra showed characteristic peaks for resin, glass filler and initiation system as seen in Figure 2, with main peak assignments provided in Table 2. 

Small variations were seen between the two unset resin pastes, with weak peaks at 1767 and 1790 cm^−1^, indicating the presence of benzyl peroxide (BPO )initiator [41]. Spectra collected from both the (i) pastes and (ii) set cements included peaks at at 1717 cm^−1^, 1637 cm^−1^, and 1608 cm^−1^, arising from the carbonyl, aliphatic and aromatic regions respectively. Peaks attributable to the borate glass filler are visible in the region of 1508 cm^−1^ (non bridging oxygen bond stretching), 1300 cm^−1^ (boron oxygen ring vibrations), and 1240 cm^−1^ (asymmetric bending of pyro- and ortho- borate groups) [40]. 

**Figure 3:**
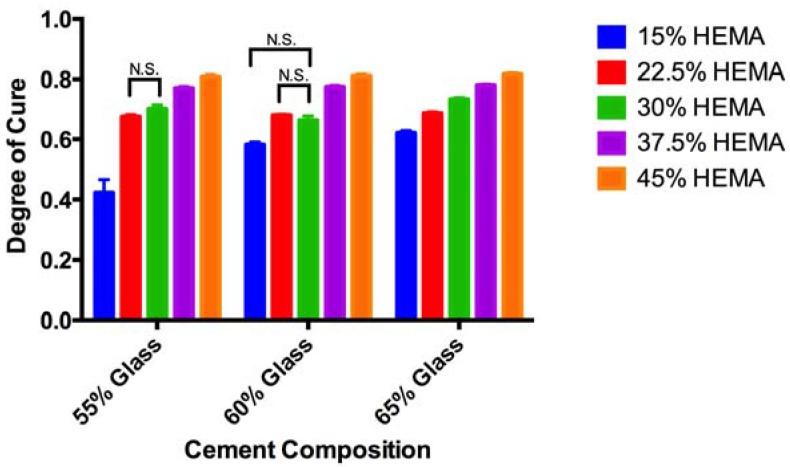
Degree of conversion measured through ATR-FTIR analysis with standard deviation, showing increased degree of conversion as the HEMA content increased, (*p* < 0.01, unless otherwise noted).

The degree of conversion for set cements (Figure 3) varied between 42% (composition A1) and 81% (composition E3). Significant increases in the degree of conversion were seen with increasing HEMA content for cements with equal glass content. Similarly, significant increases in the degree of conversion were seen between 55% and 65% glass loadings for equal HEMA contents, but not the 55% and 60%, or 60% and 65% pairing.

### 2.3. Biaxial Flexural Strength

**Figure 4:**
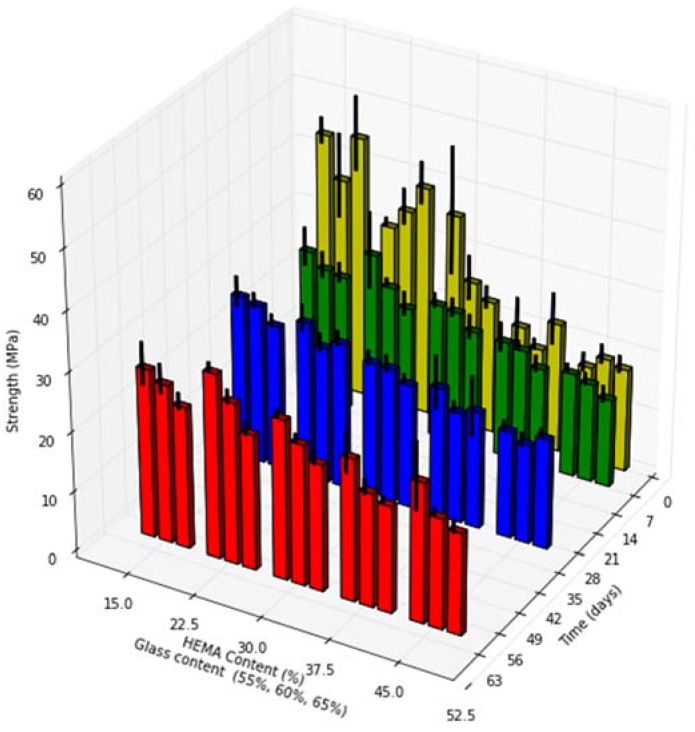
Cement biaxial flexural strength (MPa) at 1, 7, 30 and 60 days incubation, with standard deviations.

Cement biaxial flexural strength ranged from 16 MPa to 44 MPa after 24 h, and 16 to 28 MPa at the 60 day time point (Figure 4). 

Increasing HEMA content resulted in significant decreases in cement strength at all time points investigated (*p* < 0.01). Increased glass loading resulted in significant decrease in cement strength at the 60 days time point only (*p* < 0.01). A significant decrease in cement strength between the 1 and 60 days time points was seen for compositions of group A and B (*p* < 0.01), while cement compositions of group E increased in strength over 60 days (*p* < 0.01). 

### 2.4. Cement Water Sorption and Weight Loss

**Figure 5:**
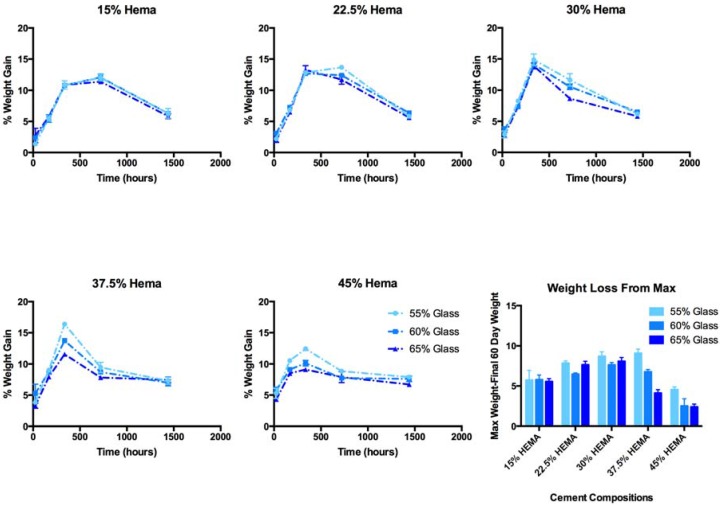
Cement weight gains over 60 days of incubation in phosphate buffered saline (PBS) (with standard deviations), and weight loss from max (calculated as difference between maximum weight gain, and final weight gain at 60 days) as a measure of glass filler loss.

After 24 h, increasing mass gain (1.3 to 5.8%) of specimens was associated with both increased HEMA and glass content in the resin composites (Figure 5). Peak mass gain occurred after 14 days for cements of group C, D and E. Conversely cements of group A and B did not achieve peak mass gain until 30 days of incubation.

Maximum mass gains ranged from 11.3% (A3, 30 days) to 16.4% (C1, 14 days) of initial dry weight. Glass loading had no effect on the long-term mass gain for compositions of group A, B and C, while glass loading exerted a significant effect for group D and E compositions. HEMA content initially resulted in significant increases in mass gain for all glass loadings; no significant effect was seen at extended time points. While sample mass decreased between 30 and 60 days, it is assumed that hydration increased, and decrease in mass is due to glass filler loss. As such, mass loss between the maximum weight achieved, and the final weight at 60 days was used as a proxy measure of glass degradation. Mass loss from peak weight gain was found to be the greatest in group C.

### 2.5. Ion Release

**Figure 6:**
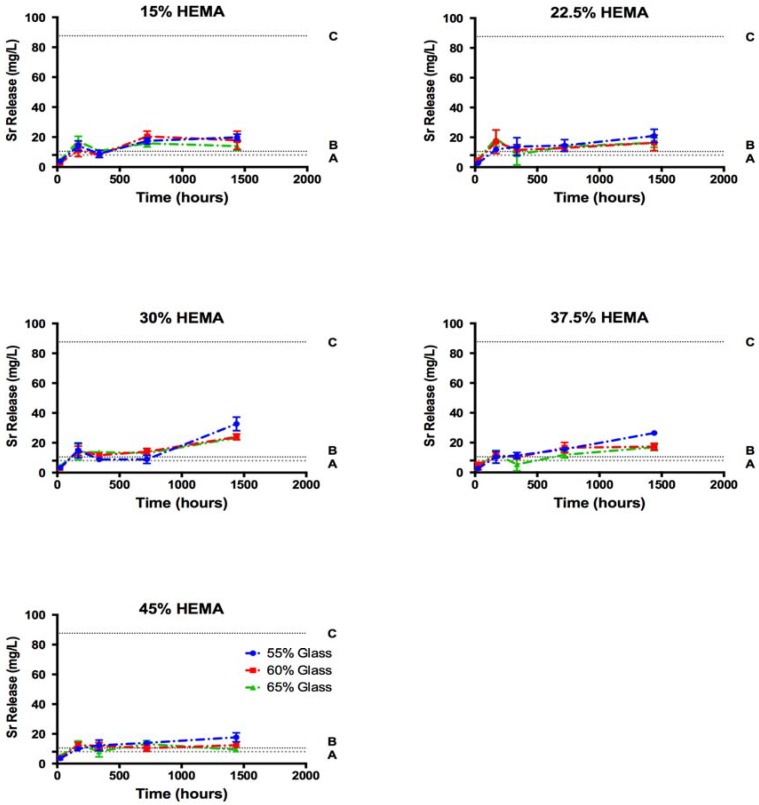
Sr release from cements into PBS in mg/L, relative to (A) in vitro osteoblast activation threshold [6]; (B) serum Sr concentration reported in the Treatment of Peripheral Osteoporosis (TROPOS) study (10.8 mg/L) [4] and (C) in vitro osteoclast inhibition threshold [15].

**Figure 7:**
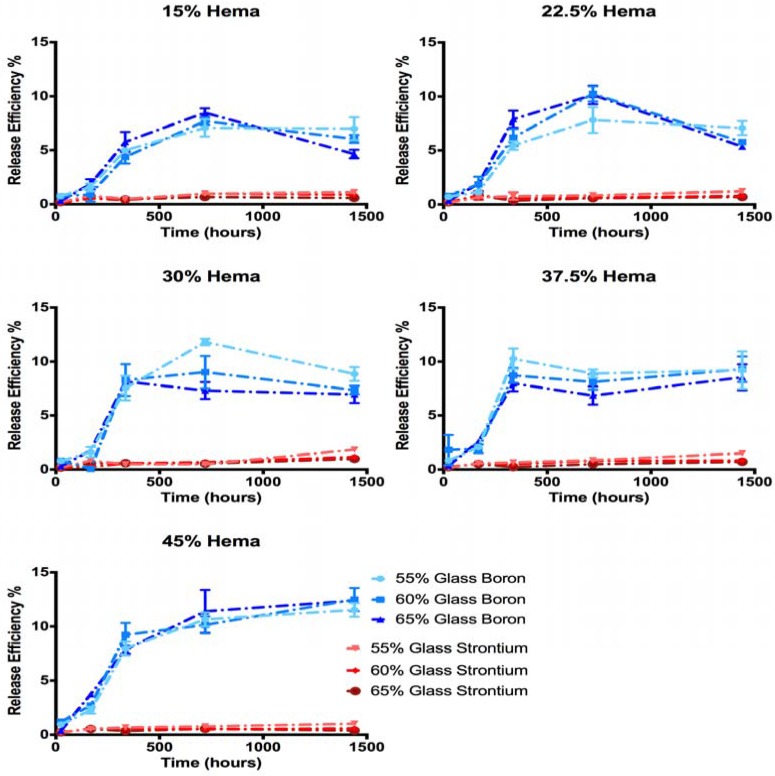
Ion release efficiency as % of initial elemental loading for B and Sr demonstrating preferential release of B from the cements.

Total Sr release from cements ranged from 9 to 32 mg/L following 60 days of incubation in phosphate buffered saline (Figure 6). These release levels corresponded to between 0.3 and 1.8% of the Sr initially present in the samples (Figure 7). B release levels were much greater ranging from 67.7 to 183.6 mg/L, the equivalent of 4.6 to 12.9% of the initial B content at the 60-day time point.

No significant differences in Sr release levels were seen between the compositions after 1, 7 and 14 days. Following 30 days of incubation cements of groups C, D and E released more Sr than group A and B samples (*p* < 0.01). After 60 days of incubation, samples group C released significantly more Sr than all other HEMA loadings (*p* < 0.01). Release efficiency of Sr, measured as the % of initial Sr loading which was released into solution ranged from 0.3 to 1.3% after 60 days, while release efficiency of B reached a maximum of 8 to 15%. Previous investigation into the dissolution kinetics of the glass filler used showed preferential release of Sr from the glass network utilized in this study (i.e., the release efficiency of Sr was greater than the release efficiency of B) [35]; conversely, the cement extract data in this study showed greater release levels for B relative to the Sr release. As B release shows the degradation of the glass network, it is assumed that Sr is in fact released concurrently from the glass during degradation, but is not free in solution. 

**Figure 8:**
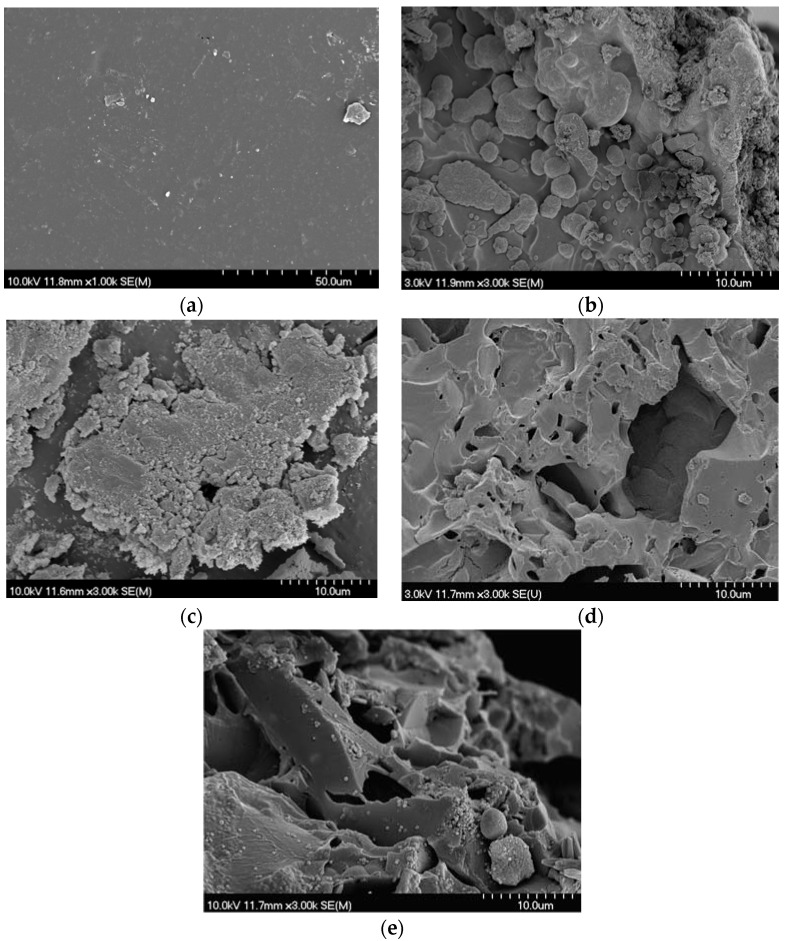
Representative scanning electron microscope images of resin surfaces for (**a**) control unreacted surface of group E, 60% glass cement (**b**) Group E 60% glass cement, 1 day of incubation in PBS; (**c**) group E 60% glass, 60 days of incubation in PBS; (**d**) group A 60% glass, 1 day of incubation in PBS; and (**e**) group A, 60% glass 60 days of incubation in PBS, demonstrating the development of a strontium rich surface precipitate during incubation.

SEM analysis confirmed large regions of a Sr- and phosphate-containing precipitate at the surface of cement samples, showing evidence of crystallization at later time points (Figure 8). After a single day of incubation, strontium phosphate precipitates were found on the surface of the glass filler for cements of group A, while group E cements displayed more dispersion of these deposits on the surface of both filler and resin phase. Energy-dispersive X-ray spectroscopy (EDS )analysis of the unreacted resin (Figure 8a) revealed a surface Sr content of 4.63% Sr by weight, while reacted surfaces had Sr contents of 18 to 33% after one day of incubation, increasing to 20 to 45% after 60 days. Sr enriched areas also showed between 6 and 10 wt% phosphate.

## 3. Discussion

The inclusion of HEMA, up to 45% of the resin weight was achieved without significant reduction of the cement setting time. While some variations in cement working time were observed, this is likely due to the subjectivity of the measurement method, as previously reported [42]. The observed setting times were below the 20 min target frequently cited in the literature as a requirement for vertebroplasty materials [27]. Nonetheless, Cortoss^®^, a two paste composite resin, is clinically utilized and has a reported setting time of 5–8 min for this indication [28]; as such the setting times reported for the experimental cements in this paper may not represent a clinical limitation. Specifically, if a similar “mix on demand” were to be utilized for the experimental cements, it may eliminate the time for hand mixing, and simplify workflow in the clinic. Should extended handling characteristics be necessary, it is known that pre-chilling the resins can extend the handling characteristics by up to 79% [43,44]; such an increase would leave the setting time of the cements investigated in this study within the range of those reported for Cortoss.

Aside from the clinical determinants of setting, the degree of conversion is an important predictor of safety and performance; both in terms of mechanical strength, and biological performance. With respect to the latter it is known that unreacted monomers serve to plasticize the polymer matrix, reducing cement strength. This was demonstrated by Ferracane Berge and Condon who reported at 75% increase in flexural strength through improved degree of conversion [39]. The remaining unreacted monomer may be leached into the surrounding environment, increasing the probability of the occurrence of harm to local tissues [45]. The degrees of conversion previously reported for Costoss^®^ range from 76 to 86% depending on storage conditions, similar to the results seen in this study, however for the latter (i.e., Cortoss^®^) measurements were performed using differential thermal analysis and not ATR-FTIR [43]. Measurements of degree of conversion may vary up to 10% between methods, with varying reports of over or underestimation [38,46]. Degree of conversion measurements in this study were much greater than those reported for adhesive resins with similar co-monomer compositions (30–60%), which showed a decreasing degree of conversion (as measured though ATR-FTIR) with increasing HEMA content, as opposed to the increase seen in this study [37]. A report of increased degree of polymerization with increased HEMA content, similar as to what was seen in this study however, was seen when comparing Bis-GMA HEMA blends to Bis-GMA, TEG-DMA, HEMA blends in the study of amorphous calcium phosphate resin composites developed by Antonnuci, where conversion rates ranged from 79.3 to 89.9 [38]. These conflicting reports have been attributed to either the lower reactivity of HEMA relative to Bis-GMA decreasing the degree of conversion [37], or alternatively, the low viscosity of HEMA imparting greater mobility allowing for greater cure [38]. While HEMA is less cytotoxic than both Bis-GMA and TEG-DMA, its smaller molecular size, single methacrylate functional group and greater hydrophilicity result in higher rates of leaching, giving it a greater impact on resin cytotoxicity in dental materials [45]. The data reported in this paper indicate that increased HEMA content may be beneficial for these materials beyond increasing hydration of the matrix; specifically we observe a high level of cure for the cements examined in this work, which will reduce the concentration of unreacted monomers available for leaching, and as a consequence may improve the biological response. 

From a mechanical performance standpoint, the biaxial flexural strengths of the compositions studied ranged from 44 down to 16 MPa (1 day), dropping to between 28 and 16 MPa (60 days). Biaxial Flexural strengths previously reported for commercially available vertebroplasty materials are significantly higher than the materials examined in this work. Previously reported biaxial flexural strengths for PMMA bone cements are 99 to 113 MPa after 1 and 30 days in simulated body fluid respectively, while strengths reported for Cortoss^®^ range from 96 MPa to 59 MPa after 1 and 30 days respectively [47]. While Cortoss’s two paste system is packaged preloaded in a mixing/extruding device, the cements in this study were hand mixed to accommodate the small sample size and large number of compositions investigated. Such hand mixing has previously been linked to increased porosity and decreased mechanical strength due to the inclusion of air pockets during mixing when compared to mechanically mixed cements, this may partially explain the lowered flexural strength [48]. The lower biaxial flexural strengths seen in this study also likely results from the substitution of HEMA a small mono-functional methacrylate for the larger Bis-GMA dimethacrylate. While high Bis-GMA content provides increased mechanical strength, the inclusion of smaller co-monomers as diluents is necessary for workable orthopedic or dental materials to reduce the viscosity of the resulting cement [49]. In this study, both TEG DMA, and HEMA served as diluents. Increased mechanical strength may be achieved for these cements through the elimination (or reduction) of TEG DMA content, allowing for increased Bis-GMA content, while maintain proper viscosity solely through HEMA addition. As high HEMA content cements demonstrated no loss of mechanical strength over time, with no appreciable effect cement setting time, and improved the degree of conversion, such a blend may provide improved long term strength and reduced cytotoxicity without compromising cement handling properties.

While no application specific standard exists for flexural strength of vertebroplasty materials, a 50 MPa minimum flexural strength is dictated by ISO 5833 for acrylic bone cements used in arthroplasty fixation. Much lower flexural strengths are required in non arthroplasty fixation cements, such as Hydroset, a calcium phosphate cement designed for void filling which has a biaxial flexure strength of 9 and 6 MPa at 1 and 30 days of incubation in a simulated body fluid [50]. The decrease in cement strength seen with time with both Cortoss and Hydroset was seen with the compositions of group A and B in this study, higher HEMA content cements however did not share this trend. The apparent ability of high HEMA content cements to maintain strength over time, despite the greater extent of glass filler degradation seen, may be explained by one of two factors: a more rapid loss of strength preventing the initial starting strength from being measured at the 24 h time point, or the formation of strontium phosphate precipitates within the resin as evidenced by SEM. While the current cement resin blend studied is unlikely to provide the water sorption required for extensive Sr deposits to form within the bulk of the material, the biaxial flexural test is sensitive to surface effects, and therefore may represent a surface strengthening effect form the mineral deposit seen [51]. 

Another interesting finding of this work were the changes in sample mass observed as a function of time. We propose that this observation encompasses three phenomena; (i) water sorption; (ii) glass dissolution (as shown through ion release); and (iii) the precipitation of Sr and phosphate mineral on the surface of the cements (as evidenced by SEM and EDS, Figure 8). The influence of these concurrent phenomena cannot be isolated based on the existing study design, and as such no mechanistic model for Sr ion release could be accurately fitted to our ion release data from the cements. The earlier onset of maximum weight gain in high HEMA content cement indicated a more rapid loss of glass filler, which was verified though the increased release of B into solution. Collares et al., studied a series of adhesive resin blends with similar HEMA content ranging from 15 to 50% of resin blend (in a Bis-GMA TEG-DMA, Bis EMA blend), and reported water sorption of 74.7 to 174.1 µg/cm^3^ following 7 days of incubation in distilled water. The 7-day weight gains observed in this study ranged from 95 to 187 mg/cm^3^, orders of magnitude greater. Possible explanations for the large difference in mass gain could be both the macroporosity imparted by the incorporation of degradable glass filler, as well as the precipitates seen on the surface of the cements leading to overestimating of water content. It is also of note that the degree of conversion seen in Collares’ study were much lower, and thus cured adhesive resins would contain a large fraction of unreacted HEMA, which is less hydrophilic than in its polymerized state, and may have lead to mass loss through leaching. 

The effect of glass filler content on weight gain varied with HEMA concentration, high HEMA compositions showed decreasing weight gain with increasing glass content, while low HEMA compositions showed little to no effect of glass filler fraction. Similar results were seen in a study of amorphous calcium phosphate reinforced resins, where it was noted that the effect of glass filler on water sorption was dictated by the relative hydrophilicity of the two phases [38]. Similarly in this study, low HEMA content cements showed no change in water sorption with changing glass content, as hydrophilicity did not differ sufficiently between the two phases. 

The relative release ratios of B and Sr seen in this study varied greatly from the release profiles reported for the glass (in isolation); the glass filler used is this study has previously been shown to exhibit preferential Sr release over 60 days (release efficiencies of 46% and 19% for Sr and B respectively) [35]. In this investigation, the release efficiency of B increased with increasing HEMA content (Figure 7), indicating that glass network hydrolysis did increase with HEMA content and water sorption, as expected. This trend however did not translate to increased Sr release into the solution. Previous investigation of the glass filler in isolation reported that (i) a single homogeneous glass phase was formed; and (ii) Sr release was governed by the diffusion of water into the glass network [38]. It can thus be assumed that as B release indicates the hydrolysis of the glass network, an equal or greater fraction of Sr must have been lost from the glass filler, despite not being measured in the solution. As B release reached between 8% and 15% of the initial B loading in the cements at the 60 day time point, it can be assumed that strontium release was equal to or greater than 8 to 15%, ten times what was seen in the solution (0.3 to 1.3% of initial Sr loading). As such, it may be reasonably concluded that at a minimum 8 to 15% of the loaded Sr (equivalent to B release levels) was release from the glass filler greatly exceeded all therapeutic thresholds, exceeding 100 mg/L after 14 days.

As B release measurements demonstrated extensive glass network dissolution, which conflicted with Sr release measurement through ICP-OES, SEM imaging was performed to ascertain the fate of the Sr released from the glass. SEM and EDS analysis of cement surfaces showed the development of large amounts of a Sr and phosphate-containing precipitate, which was not previously seen during the dissolution of the glass filler phase in isolation, in which Sr release concentration of up to 1127 mg/L were reported without deviation from Fickian diffusion kinetics [35]. This finding indicated that the discrepancy between release efficiency of Sr and B from the cements was the result of Sr being chelated in the form of a precipitate from solution following glass filler degradation. The formation of this precipitate would be favored not only by the mass transport constraints enforced by the resin phase of the cement, but also the polarizable oxygen groups present in the resin phase due to the high HEMA contents. Previous studies into the calcification of copolymer blends in abiotic solutions have shown increases in the rate of calcification with increasing polar oxygen pendant groups, specifically increasing HEMA content [52]. In the current study, it is likely that the increase in resin polar oxygen severed to chelate Sr from solution, generating nucleation sites for phosphate in a similar manner, resulting in the formation of the precipitate in the resin phase. While increasing HEMA content effectively increased resin hydration and served to increase B release, the concurrent increased chelation of Sr reduced the efficacy of Sr release. Due to these conflicting mechanisms, maximum Sr release was achieved in the cements of group C.

The formation of the Sr phosphate precipitates may be further exasperated by the incubation conditions selected. As calcium phosphates have a lower degree of solubility than Sr phosphates, the chelation of Sr may be artificially enhanced due to the use of a calcium free incubation media. Furthermore, Sr phosphates have been demonstrated to undergo spontaneous ion exchange substitutions to form stable calcium phosphates in calcium rich environments. Through such a mechanism it is possible that these precipitates may provide both enhanced cellular attachment, and prolonged Sr release through ion exchange. Testing of these materials under more dynamic conditions, in a calcium-rich environment, may provide information into the stability of this precipitate, and the potential of this layer to act (i) as a reservoir of Sr or as (ii) a site for cellular attachment. 

Overall the Sr release from the materials developed and investigated in this study ranged between 9–32 mg/L after 60 days of incubation; falling within the range previously reported for increased osteoblast activity, but well below that of the osteoclast inhibition threshold in vitro. A study of Sr substituted dicalcium phosphate dihydrate cements at loading of up to 8% of cement weight allowed for Sr release levels of 20 mg/L over 3 weeks, similar to levels seen in this work [25]. While the Sr-containing calcium phosphate cements demonstrated improved cell proliferation and alkaline phosphatase (ALP) expression over plastic tissue culture plates, they did not offer an advantage over purely calcium-based controls. Schumacher et al. reported maximum Sr release levels of 13 mg/L from Sr substituted calcium phosphate cements, and was able to demonstrate a significant increase in osteoblast proliferation and differentiation in comparison to a calcium phosphate control at these release levels, with indirect cell culture methods [53]. These previous results may indicate that these release levels in this study are sufficient for in vitro efficacy, however the development of the precipitate masked our ability to determine total Sr release. Based on the present data, the Sr release levels observed are likely insufficient for an uncoupling effect in vitro. 

The serum Sr levels required for the uncoupling effect are much lower than those required in vitro (10.5 mg/L) which may be a result of the systemic effect of Sr through the parathyroid gland, or the cell culture methods used. While osteoblast osteoclast communication is known to play an important role in the control of bone metabolism, in vitro efficacy levels have largely focused on monoculture conditions, leaving true localized in vivo requirements for uncoupling unclear. Serum Sr concentration required for uncoupling are achieved by many of the cements investigated (all but group E), and as such may have some beneficial effect towards uncoupling of bone turn over. Furthermore, it is possible that the use of calcium-rich media may prevent Sr chelation, increasing Sr release up to tenfold, well above the 88 mg/L reported for the inhibition of osteoclast activity. Further investigation into the effect of incubation media on Sr release, and the in vitro efficacy through dual culture conditions could provide guidance for the further development of materials designed for the local release of Sr.

## 4. Materials and Methods

### 4.1. Cement Fabrication

**Figure 9:**
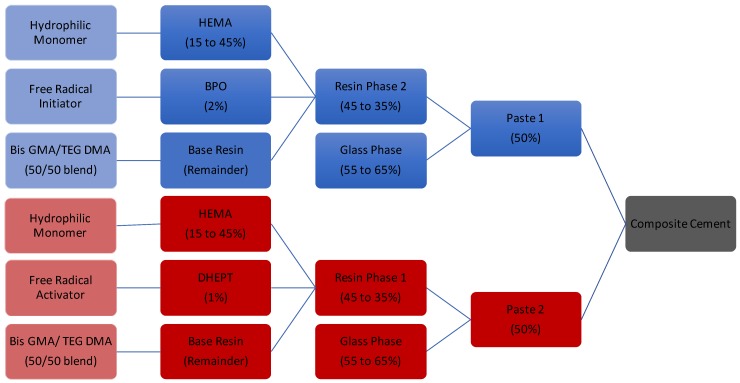
Schematic of two paste resin fabrication, all compositions represented in weight percentage.

Borate glass containing 70% boron oxide, 26% strontium oxide and 4% sodium oxide (mole fraction) was synthesized as per MacDonald et al., and sieved to retrieve <25 μm particles [35]. Particle size distribution was verified using a laser particle size analyzer, in aqueous dispersant (Mastersizer 3000, Malvern, Malvern, Worcs, UK), and confirmed as d_10_ = 3.38 μm, d_50_ = 12.1 μm, d_90_ = 26.4 μm. A series of 15 two-paste resins (Figure 9, Table 1) were fabricated through varying mixtures of HEMA (Aldrich Chemistry, USA), TEGDMA (Aldrich Chemistry, St. Louis, MO, USA), and BisGMA (Aldrich Chemistry, USA). Benzyl peroxide (BPO, Sigma Aldrich, St. Louis, MO, USA) and *N*,*N*-Bis(2-hydroxyethyl)-*p*-toluidine (DHEPT, Aldrich Chemistry, St. Louis, MO, USA) were added to paste one and two respectively, for each composition, to form a chemical initiation system [54]. Resin components were shielded from light to prevent photo initiation of benzoyl peroxide, and placed in a shaking incubator at (37 °C, 2 Hz) overnight to reduce resin viscosity and allow for mixing. Resin mixtures were refrigerated until further use, and hand spatulated with the appropriate weight of glass to form both paste components as needed. 

### 4.2. Cement Handling Characteristics

Cement working times were assessed in triplicate as described by Dickey et al. [55]. Cement setting time was determined to be equal to the time to reach 50% of the exothermic peak following the mixing of the two pastes, as per established international protocols [56]. Dimensions of the curing mold used were reduced to 30 mm diameter and 15 mm height, to reduce the material needed for testing to 10 g/sample. 

### 4.3. Degree of Conversion

ATR-FTIR analysis was performed on both ‘unset’ and ‘set’ cements to assess the degree of conversion via observations of the relative decrease in the aliphatic carbon-carbon double bond (C=C) peak at a wavenumber of 1638 cm^−1^. Set samples were generated through hand spatulation on a dental pad using equal amounts (1 g) of pastes one and two. Subsequently, cements were transferred into polytetrafluoroethylene (PTFE) molds (7 mm ø × 1 mm), sandwiched between acetate sheets, and clamped between steel plates to set a room temperature for 1 h [57]. The cement samples were then scrapped of excess material (i.e., flash), removed from molds and stored for 24 h in a desiccator prior to analysis. Three discs were produced for each cement composition with two measurement locations used for each disc (*n* = 6). Unset cement spectra were collected through measurements of individual paste components (*n* = 3 per paste, total *n* = 6 per cement composition). ATR-FTIR measurements were performed on a TENSOR 27 system (Bruker, Billerica, MA USA) using a diamond attenuated total reflectance (ATR )crystal (Golden Gate, Specac, Orpington, Kent, UK). Spectra were collected as the average of 6 scans, between 1200 cm^−1^ and 1800 cm^−1^ at a resolution of 4 cm^−1^. To assess the stability of the individual pastes 500 spectral scans were gathered over 120 min. Degree of conversion for all samples was calculated through the ratio of absorbance peaks centered at 1638 cm^−1^ and 1608 cm^−1^, representative of the unreacted methacrylate functional groups and aromatic rings of the Bis-GMA respectively [58]. For each cement composition, the degree of conversion was calculated as the relative ratios of the methacrylate peak area before and after curing normalized to the aromatic peak area, using Equation (1);

(1) DC = 1−[(AmAa)set(AmAa)unset]

where ‘Am’ is the area of the methacrylate peak (1638 cm^−1^) and ‘Aa’ is the area of the aromatic control peak (1608 cm^−1^) [39].

### 4.4. Biaxial Flexural Strength

Cement disks (15 mm ø × 1 mm) were fabricated in PTFE molds, and allowed to cure for 1 h in ambient conditions [57]. Following removal from the molds the cement disks were placed in PBS (35 mL/sample), and incubated in a shaking incubator at 37 °C, 2 Hz for 1, 7, 30 and 60 days (*n* = 5 per condition). Following incubation, each sample was patted dry, measured in duplicate for diameter (accuracy of 0.1 mm) and mounted in a ball on 3 ball biaxial flexural testing rig. Testing was undertaken using an Instron 3400 (Instron, Norwood MA, USA) system fitted with a 2 kN load cell at a strain rate of 1 mm/min. Sample thickness was measured in triplicate following sample rupture at the crack origin. Fracture loads were converted into biaxial flexure strengths using the methods described by Chung et al., using Equations (1) and (2).

(2) $$\sigma_{biaxial}\;=\;\frac{3P\left(1\;+\;\nu \right)}{4\pi t^{2}}\left[1\;+\;2\ln\frac{a}{r_{ο}^{*}}+\;\frac{1\;-\;\nu \;}{1\;+\;\nu }\left(\frac{2a^{2}\;-\;r_{ο}^{*2}}{2c^{2}}\right)\right]$$

(3) $$r_{ο}^{*}\;=\;\sqrt{\left(1.6r_{ο}^{2}\;+\;t^{2}\right)\;}-\;0.675t$$

*P* is the load at failure, *v* is the Poisson’s ratio, (assumed to be 0.24), t is sample thickness, *a* is the radius of the support circle, *c* is the radius of the sample, and *r_o_* is the radius of central loading ball [51].

### 4.5. Cement Water Sorption and Weight Loss

Cement samples were generated through hand spatulation on a dental pad using equal amounts (1.5 g) of pastes one and two. Subsequently, cements were transferred into stainless steel split ring cylindrical molds (4 mm ø × 6 mm), sandwiched between acetate sheets, and clamped between steel plates to set a room temperature for 1 h [57]. The cement samples were then scrapped of excess material, removed from molds, weighed for dry mass, placed in 10 mL (1 cm^2^/10 mL) of phosphate buffered saline, capped, and stored in a shaking incubator (37 °C, 2 Hz) for 1, 7, 14, 30, or 60 days respectively (*n* = 3/time point). Following incubation samples were removed using a disposable spatula, patted dry, and wet weights were recorded. Subsequently, the extract medium was capped and stored in a refrigerator until ion release analysis. 

### 4.6. Ion Release Profiles

Extracts generated through the cement water sorption studies over 1, 7, 14, 30 and 60 days were decanted, and diluted in 2% ultrapure hydrochloric acid (Fluka, Mexico City, Mexico) for ICP analysis of Sr and Boron (B) content (Na measurement omitted due to high background measurements in PBS) [35]. Inductively Coupled Plasma optical emission spectroscopy analysis was performed using an Optima 8000 (Perkin Elmer, Waltham, MA, USA) with a 2% hydrochloric acid suspension media, nitrogen carrier gas and an argon flame. Calibration curves were produced using certified Perking Elmer Pure ICP standards (Perkin Elmer, Waltham, MA, USA). All samples were measured in triplicate and recorded as the mean of 3 readings. All ion release measurements were reported in mg/L for assessment of therapeutic potential, and normalized to initial sample content for assessment of degradation kinetics.

### 4.7. SEM Analysis of Weight Loss Samples

SEM images were collected for 15% HEMA and 45% HEMA samples following 1 and 60 days of incubation in phosphate buffered saline. Samples were sputter coated with a gold-palladium coating using a Lecia EM ACE200 (Wetzlar, Germany). BS-SEM images were collected using an Hitachi S4700 FED (Hitachi, Chula Vista, CA, USA) operating at 3 kV, 15 µA, under 30 time magnification, or 3 kV, 16.5 µA, 250 times magnification.

### 4.8. Statistical Analysis

Statistical analysis was performed using Prism 6 (GraphPad Inc., LaJolla, CA, USA) for the two-way analysis of variance (ANOVA) (assessing effect of HEMA and glass content) with a Tukey multiple comparison test, at a significance level of 0.05. Time effects were analyzed using a two way ANOVA, with repeated measures, with a Tukey multiple comparison test. Peak fitting of Fourier transform infrared (FTIR) data was performed using Python (Python Software Foundation, Wilmington, DE, USA). 

## 5. Conclusions

Increasing the HEMA content of the cements led to an increase in mass gain and degree of cure without significantly effecting cement setting time. While filler network hydrolysis increased with increased water sorption (as evidenced through increased B release), the relative release ratio of Sr to B dropped with increasing HEMA content. It was confirmed that this observation was associated with precipitation of a Sr phosphate phase on the surface of the cements, suggesting an interaction between free Sr and the resin phase under the experimental conditions utilized. The concentration of Sr required for in vitro osteoclast inhibition was not seen under the study conditions. However, the materials did release and maintain Sr at the levels required to increase osteoblast proliferation. Further investigation into the effect of incubation conditions on the rate of Sr release (namely the effect of calcium in the incubation media, and mechanical loading) may provide further insight into the mechanism of ion release from such materials, and allow for more accurate extrapolation into in vivo conditions.

## Figures and Tables

**Table 1:** Compositions of resin phase, and glass loading of the cement compositions, in weight percentage.

Composite		Group A			Group B			Group C			Group D			Group E		
		1	2	3	4	5	6	7	8	9	10	11	12	13	14	15
Resin Phase Part One	Bis-GMA	41.474			37.725			33.975			30.225			26.475		
	TEGDMA	41.475			37.725			33.975			30.225			26.475		
	**HEMA**	**15**			**22.5**			**30**			**37.5**			**45**		
	BPO	2			2			2			2			2		
Resin Phase Part Two	Bis-GMA	42			38.25			34.5			30.75			27		
	TEGDMA	42			38.25			34.5			30.75			27		
	**HEMA**	**15**			**22.5**			**30**			**37.5**			**45**		
	DHEPT	1.00			1.00			1.00			1.00			1.00		
**Glass**		**55**	**60**	**65**	**55**	**60**	**65**	**55**	**60**	**65**	**55**	**60**	**65**	**55**	**60**	**65**

**Table 2:** ATR-FTIR peak locations with corresponding cement component, and functional group.

Band Region (cm^−1^)	Cement Component	Functional Group	Reference
**1700–1720**	Resin	C=O and C=O hydrogen bonding	[38]
**1630–1640**	Resin	C=C double bonds	[39]
**1600–1610**	Resin	Aromatic Rings	[39]
**1500–1520**	Glass	Non Bridging oxygen stretching	[40]
**1280–1320 (2 peaks)**	Glass	Boron-oxygen ring vibrations	[40]
**1240**	Glass	Ortho and Pyro borate asymmetric bond stretching	[40]
**1767 & 1790**	Initiator	Benzyl Peroxide Ring Vibration	[41]
